# Tolerability of immunoglobulin products and outcomes after product switching: a single-center real-world retrospective study

**DOI:** 10.3389/fimmu.2026.1870107

**Published:** 2026-08-14

**Authors:** Paulina Vásquez-Reyes, Andrea Ferranti-Ramos, Daniel Iguasnia Portilla, Helena Fernández-Cabrera, Isabel Castillo-Castillo, Oana Irina Sobieschi, Luis Fernandez Pereira

**Affiliations:** Clinical Immunology and Molecular Genetics Unit, Hospital Universitario San Pedro de Alcántara, Cáceres, Spain

**Keywords:** adverse drug reaction, immunoglobin, intravenous, subcutaneous, switching

## Abstract

**Objectives:**

We assessed the frequency of adverse drureactions (ADRs) to immunoglobulin products, associated switching patterns, and tolerability after switching in clinical practice.

**Methods:**

This observational, retrospective study was conducted at a single center. We included all patients who were treated with immunoglobulin therapy, irrespective of indication, between June 2017 and May 2025. Data on demographics, diagnoses, immunoglobulin received, ADRs, need for switching, and post-switch ADRs were extracted from records. Analyses were mainly descriptive.

**Results:**

We included 51 patients with a median (Q1, Q3) age of 54 (40, 63) years, mostly with primary or secondary immunodeficiencies, who were treated with intravenous immunoglobulins (IVIGs; n = 43) or subcutaneous immunoglobulins (SCIGs; n = 8). Among 42 patients treated with intravenous (IV) Flebogamma^®^, 22 (52%) experienced ADRs, and 12 (29%) required switching. No ADRs were observed after switching, regardless of whether patients switched to another IVIG or SCIG. Among the seven patients treated with facilitated SCIG (SC HyQvia^®^), three experienced ADRs, and one of them was switched back to conventional SCIG (SC Hizentra^®^) due to persistent local adverse reactions, with no further ADRs reported after the switch.

**Conclusion:**

ADRs associated with IVIG are common and lead to treatment switching in a high proportion of patients. Switching to an alternative immunoglobulin product appears to be a good alternative for managing ADRs.

## 1 Introduction

Immunoglobulin therapy is well established for primary and secondary immunodeficiencies, as well as certain autoimmune disorders (1). The various intravenous immunoglobulin (IVIG) products are considered equally effective for treating these conditions (2). However, the tolerability and safety of IVIG differ across products depending on the formulation, stabilizers, osmolality, IgA content, and excipient profiles; thus, each product should be considered unique and should not be considered interchangeable from a tolerability perspective (3, 4). Since the 1990s, subcutaneous immunoglobulin (SCIG) use has increased and demonstrates comparable efficacy to IVIG and a distinct safety profile characterized by fewer systemic reactions but more frequent local reactions (5–7).

Although generally considered safe, immunoglobulin replacement therapy is associated with adverse reactions, which, although sometimes unpleasant or uncomfortable, are usually mild and reversible (4, 8, 9). However, severe and serious adverse reactions have also been described, including anaphylactic reactions, aseptic meningitis, renal impairment, thrombotic events, and hemolysis (4, 8, 9). SCIGs usually show better tolerability than IVIGs, with local reactions occurring more frequently with SCIGs and systemic reactions with IVIGs (3, 7, 8). The management of adverse drug reactions (ADRs) associated with immunoglobulin replacement therapy includes strategies such as slowing the infusion rate, premedication, specific treatment of adverse reactions, or switching to another preparation (3). Although frequently used in clinical practice (10, 11), the frequency and outcomes of switching among immunoglobulin products due to adverse reactions remain poorly studied (12, 13).

In this retrospective study, we aimed to assess the frequency of adverse reactions to immunoglobulin products, associated switching patterns, and tolerability after switching in clinical practice.

## 2 Materials and methods

This observational, retrospective study was conducted at a single center and approved by the Research Ethics Committee for Medicines of Cáceres (*Comité de Ética de la Investigación con medicamentos de Cáceres*, Cáceres, Spain). Written informed consent was obtained from all participants.

We included all patients treated with immunoglobulin products, regardless of indication, between June 2017 and May 2025.

Data were extracted from medical records, including demographics, diagnoses, immunoglobulin received, ADRs experienced with the immunoglobulin, need for switching, and post-switch ADRs.

The statistical analysis was mainly descriptive in nature. Quantitative variables were described using the mean and standard deviation, or the median and first and third quartiles; qualitative variables were described using absolute and relative frequencies. An exploratory bivariate analysis was performed to assess the factors associated with the occurrence of adverse reactions; continuous variables were compared using Student’s t-test, and qualitative variables were compared using the chi-square test.

## 3 Results

We included 51 patients with a median (Q1, Q2) age of 54 (40–63) years, who were predominantly male (70%), who had mostly primary or secondary immunodeficiencies, and who had initially received IVIG (**Table 1**).

**Table 1:** Demographics and clinical characteristics.

Characteristic	N = 51
Age (years), median (Q1, Q3)	54 (40–63)
Sex (male), n (%)	36 (70.6)
Diagnosis, n (%)	
Primary immunodeficiency	22 (43.1)
Secondary immunodeficiency	20 (39.2)
Other diagnoses	9 (17.6)
Type of immunoglobulin, n (%)	
Intravenous	43 (84.3)
Subcutaneous	8 (15.7)

**Figure 1:**
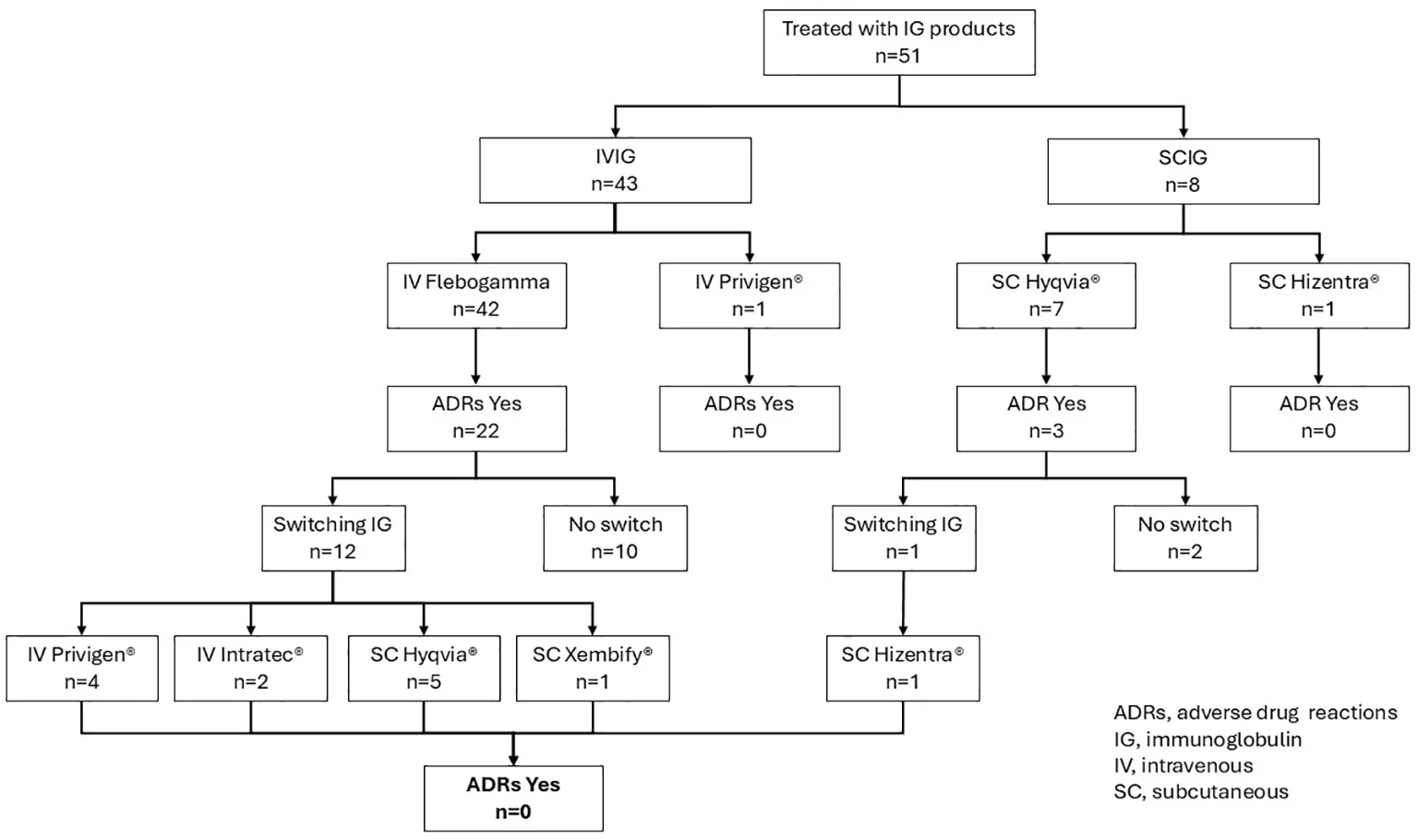
Flowchart of patients treated with immunoglobulins, occurrence of adverse drug reactions, switching patterns, and tolerability outcomes.

**Figure 2:**
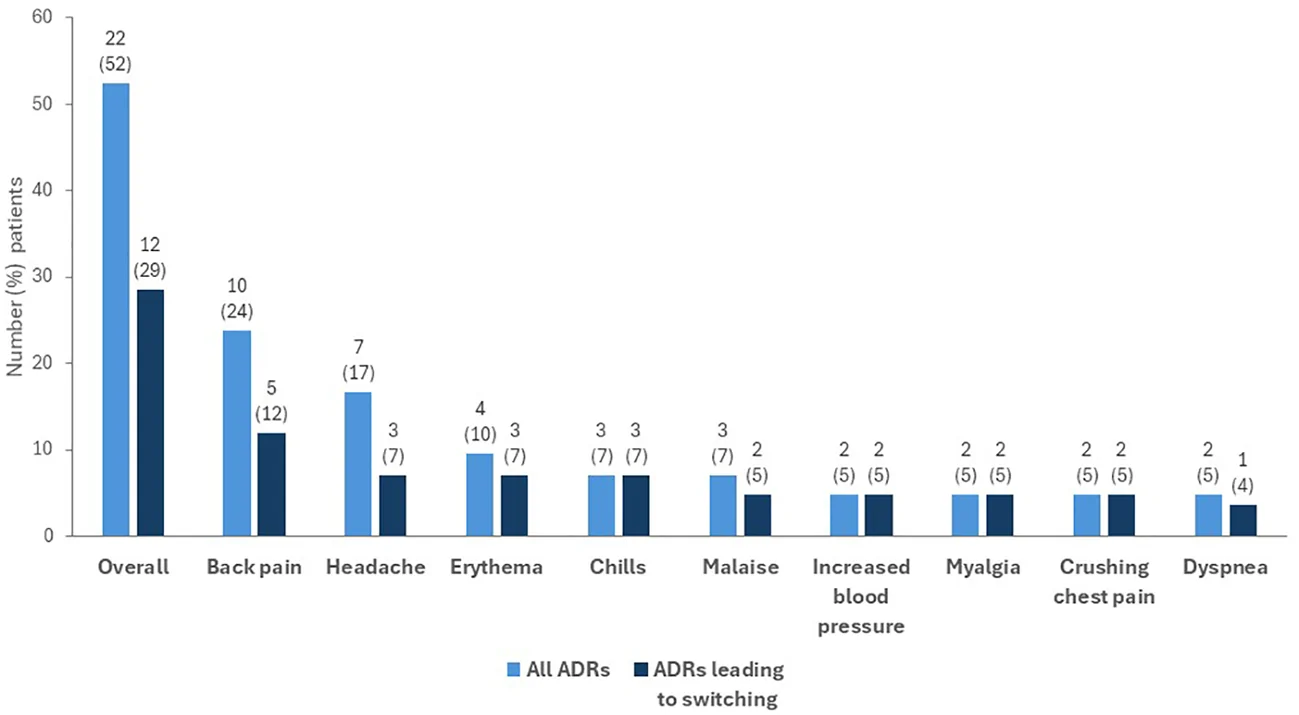
Adverse drug reactions with IV Flebogamma^®^: overall (≥5%) and those leading to switching. Patients could have experienced more than one adverse reaction. Percentages were calculated over the total number of patients who received IV Flebogamma^®^ (n = 42) and were rounded. *Other adverse drug reactions experienced by one patient each were cervicalgia, abdominal pain, ocular pruritus, fever, dizziness, and palpitations. **Other adverse reactions leading to switching experienced by one patient each were cervicalgia, ocular pruritus, fever, and palpitations.

Among the patients who received intravenous (IV) immunoglobulin replacement therapy (n = 43), 42 received IV Flebogamma^®^, and 1 received IV Privigen^®^ (**Figure 1**). Twenty-two patients (52%) treated with IV Flebogamma^®^ experienced ADRs (**Figure 2**): 14 reported mild ADRs, and 8 reported moderate ADRs. The most frequent ADRs to IV Flebogamma^®^ were back pain (n = 10, 24%), headache (n = 7, 17%), and erythema (n = 4, 10%) (**Figure 2**). In the bivariate analysis, age was the only factor associated with the occurrence of ADRs to IV Flebogamma^®^ (**Table 2**).

**Table 2:** Bivariate analysis of the factors associated with the occurrence of adverse drug reactions (IV Flebogamma) (n = 42).

Characteristic	With ADRs to IV Flebogamma^®^ n = 22	Without ADRs to IV Flebogamma^®^ n = 20	P-value
Age (years), mean (SD)	58.3 (11.5)	48.2 (13.9)	0.013
Sex			
Male	5 (22.7%)	8 (40%)	0.227
Female	17 (77.3%)	12 (60.0%)	
Diagnosis			
Primary ID	8 (36.4%)	8 (40.0%)	0.523
Secondary ID	11 (50.0%)	7 (35.0%)	
Other	3 (13.6%)	5 (25.0%)	
Comorbidities			
Yes	19 (86.4%)	14 (70.0%)	0.197
No	3 (13.6%)	6 (30.0%)	

ADRs, adverse drug reactions: ID, immunodeficiency; IV, intravenous; SD, standard deviation.

Among the IV Flebogamma^®^-treated patients, 12 patients (29%) experienced ADRs that prompted switching to another immunoglobulin (**Figure 1**). The most frequent ADRs leading to switching were back pain (n = 5, 12%), headache (n = 3, 7%), erythema (n = 3, 7%), and chills (n = 3, 7%) (**Figure 2**); all patients who reported chills, increased blood pressure, myalgia, and crushing chest pain were switched to another immunoglobulin product (**Figure 2**). After switching from IV Flebogamma^®^, none of the 12 patients experienced recurrent ADRs, regardless of whether they were switched to another IVIG (most commonly IV Privigen^®^) or to SCIG (most commonly HyQvia^®^) (**Figure 1**). In bivariate analysis, older age was the only factor significantly associated with ADR occurrence; sex, diagnosis, and the presence of comorbidities were not associated (**Table 2**).

Among the seven patients treated with facilitated subcutaneous immunoglobulin (SC HyQvia^®^), local reactions included edema (n = 2) and erythema (n = 2), as well as isolated cases of a burning sensation, myalgia, and pruritus. Importantly, two patients developed deep necrotic skin ulcers at the injection site while receiving SC HyQvia^®^. One of these cases occurred in a 33-year-old woman diagnosed with common variable immunodeficiency, without comorbidities, and the other occurred in a 25-year-old woman with a history of idiopathic thrombocytopenic purpura who had previously been treated with IV Flebogamma^®^. In both patients, the ulcers resolved and did not recur after the needle length was reduced from 9 to 6 mm. Additionally, one patient receiving SC HyQvia^®^ was switched back to conventional SCIG (SC Hizentra^®^) because of persistent local adverse reactions, and no further ADRs were reported after the switch.

## 4 Discussion

The frequency of adverse reactions to IVIG (IV Flebogamma^®^ in our series) was high (52%). This rate exceeds that reported in a postmarketing authorization study conducted in 19 centers in Spain, the United Kingdom, and Germany, which reported that 12 of 69 patients (17%) experienced adverse events potentially related to the study product (14). However, direct comparison is limited because in the postmarketing authorization study, children and adults were included. In a review of the literature, Cherin et al. (4) reported a large variation across the studies, with 1% to 81% of patients or infusions reporting ADRs. As expected, most ADRs were mild, and the tolerability profile was consistent with that described for IV Flebogamma^®^ (15); thus, from the most frequent adverse reactions reported in our study, only erythema (10%) and dyspnea (5%) were considered uncommon (i.e., a frequency <1%) according to the IV Flebogamma^®^ package insert (15). Although only exploratory, our bivariate analysis revealed that the patients with adverse reactions were significantly older. Older age is one of the factors associated with the occurrence of adverse reactions to immunoglobulins (8), and some management strategies have been proposed to minimize adverse reactions to IVIG in patients of advanced age (16).

Despite the mild-to-moderate severity of ADRs, 29% of the patients treated with IVIG were switched to another immunoglobulin product because of adverse reactions. Regardless of the adverse reactions that led to switching and whether patients were switched to another IVIG or SCIG, switching was well tolerated in all cases. These results are consistent with the limited published evidence. Feldmeyer et al. (13) conducted a retrospective study in Switzerland for a variety of indications, but mostly hypogammaglobulinemia, cytomegalovirus, or Epstein–Barr virus mismatch in lung transplant recipients and toxic epidermal necrolysis; 13 of the 40 patients (33%) experienced mild-to-moderate adverse reactions during the administration of an IV normal human immunoglobulin, which did not recur after switching to an alternative IV preparation.

Skin necrosis has been reported following SCIG (11, 17, 18). In our series, we reported a case of deep necrotic skin ulcers that was resolved after the needle length was switched from 9 to 6 mm.

Our study has certain limitations, including its retrospective design, which restricts the availability of data, and the fact that we did not record efficacy data. Despite these limitations, our results suggest that ADRs in patients who receive IV immunoglobulins are common and lead to switching to another immunoglobulin product in a high proportion of patients. Switching appears to be a good alternative for managing the ADRs associated with the use of intravenous immunoglobulins.

## Data Availability

Data are available from the corresponding author upon reasonable request. This request must include a methodologically proposal and will be evaluated in accordance with General Data Protection Regulation [GDPR] or equivalent local legislation, ethical approvals, institutional policies, and data sharing agreements.
